# The Role of Substance Use Disorders on Suicidal Ideation, Planning, and Attempts: A Nationally Representative Study of Adolescents and Adults in the United States, 2020

**DOI:** 10.1177/11782218231216233

**Published:** 2023-12-17

**Authors:** Abenaa A Jones, Gregory Hard, Joy Gray, Hannah B Apsley, Alexis R Santos-Lozada

**Affiliations:** 1 Department of Human Development and Family Studies, Pennsylvania State University, University Park, PA, USA; 2 Consortium on Substance Use and Addiction, Penn State University, University Park, PA, USA; 3 Elara Group, Las Vegas, NV, USA; 4 Department of Educational Psychology, Counseling, & Special Education College of Education, Pennsylvania State University, University Park, PA, USA

**Keywords:** Suicide, substance use disorder, adolescents

## Abstract

Few nationally representative studies examine suicidality and substance use during 2020; as such, we explored the role of substance use disorders (SUDs) on suicidality among adults and adolescents in 2020. Data were derived from N = 26,084 adult participants, representing 240 million U.S. adults weighted, and N = 5,723 adolescent participants, representing 25 million U.S. adolescents (12-17 years.). Separate logistic regressions for adults and adolescents were used to assess the association of DSM-5 SUDs, related factors, and suicidal thoughts and behaviors (ideation, planning, and attempts). In 2020, adults with SUDs were nearly 4 times more likely to seriously consider suicide (aOR = 3.94, 95% CI: 3.19, 4.86), 3 times more likely to make a suicide plan (aOR = 3.09, 95% CI: 2.25, 4.25), and nearly 4 times more likely to attempt suicide (aOR = 3.77, 95% CI: 2.29, 6.19) than adults without SUDs. Adolescents with SUDs were 4 times more likely to consider suicide (aOR = 3.69, 95% CI: 2.47, 5.51), 5 times as likely to make a suicide plan (aOR = 5.14, 95% CI: 3.25, 8.13) and to attempt suicide (aOR = 5.27, 95% CI: 2.91, 9.53) than adolescents without SUDs. Adult females and individuals experiencing poverty were twice as likely to attempt suicide than adult males and individuals not living in poverty. Adolescent females were 3-5 times more likely to seriously consider, plan, and attempt suicide than adolescent males. Interventions to curb suicidality among individuals with SUDs are crucial.

## Introduction

### Substance use and suicidality in 2020

In 2020, more individuals initiated substance use or increased their use of substances to cope with negative emotions during the COVID-19 pandemic,^1,2^ and drug overdose deaths increased by 31% in the United States compared to 2019.^3^ Pandemic-related shifts in drug availability, such as reducing the availability of drugs of choice, may have led to the proliferation of other, more potent drugs, such as fentanyl, potentially increasing adverse outcomes.^4,5^ Along with an exacerbated drug overdose death rate in 2020, concerns regarding population and individual-level psychological distress compounded. Around 46 000 individuals died by suicide in 2020, rising nearly 30% since 2000.^6^ While there was a slight overall decrease in completed suicides in 2020 compared to 2019, other aspects of suicidality, such as suicidal ideation and attempts and other related mental health concerns during the COVID-19 pandemic, are still of concern.^2,6-9^ Killgore et al^10^ found that the number of individuals who reported suicidal ideation increased each month while a state remained in “lockdown” status, with no such increase found in areas without active lockdown policies. Similarly, Hawton et al^11^ found a similar relationship between the introduction of COVID-19 lockdown restrictions and hospital visits for self-harm with the strongest effect among females.^11^

At the intersection of mental health and substance use, there is substantial comorbidity between SUDs and most forms of psychopathology.^12,13,14^ In the years preceding the pandemic, marked declines in self-reported mental and physical health were noted, and the overall increases in substance use and overdose deaths compound concerns.^15,16^ Co-occurring SUDs in individuals with a psychiatric diagnosis increase the risk for suicidal ideation and attempts, and SUDs may predict an earlier onset and greater severity of psychiatric symptoms.^17^ Substance use may also be an independent risk factor for suicidal ideation and attempts; suicides are a leading cause of death among those with substance use disorders (SUDs), particularly due to the disinhibition involved with intoxication.^18^ Research shows that opioids are involved in 20% of suicides; other drugs such as marijuana (10%), cocaine (5%), and amphetamines (3%) are also implicated in suicides, albeit at a lesser prevalence.^18^ In addition, those who use multiple substances are at even higher risk of suicidality and other negative psychosocial outcomes.^12,13,19,20-22^

### Substance use, suicidality, and vulnerable groups

The rise in deaths from completed suicides and drug overdoses is implicated partially in the recent decreases in overall life expectancy in the United States.^13,23^ Yet, suicidality and SUDs disproportionately impact younger adults and adolescents.^24,25^ Excluding unintentional causes of death, suicide is the second leading cause of death among adolescents, particularly among adolescents aged 14-18.^24^ Recent research comparing emergency department visits for suicide attempts before and during the pandemic found a 50% increase in suicide attempts among female adolescents aged 12-17; less than a 4% increase was noted for male adolescents.^26^ Regarding substance use, finding from Niles et al^27^ suggest a 94% increase in overdose deaths among adolescents from 2019 to 2020, largely due to increases in fentanyl use.^28^

### Current study

Current studies on suicidality focus on adolescents or adults, do not include the year 2020, measure only an aspect of suicidality, or do not assess the role of SUDs on suicidality.^21,22,24,26^ Few studies assess substance use disorders (SUDs) and suicidality factors within nationally representative data or examine rates of suicidal thoughts and behaviors among adults and adolescents simultaneously during the pandemic. In this study, we explored the relationship between substance use disorders and suicidal thoughts and behaviors among adults and adolescents during the pandemic year of 2020. Moreover, we assess age-specific correlations (eg, adults and adolescents separately), economic (eg, poverty level), and social factors (eg, race/ethnicity) and their relationship with suicidality during the pandemic year. We focus on 2020 as a unique year globally and nationally, with significant increases in substance use to cope with global uncertainty, limited availability of substance use treatment and mental health treatment in general, increased social isolation, and record-breaking drug overdose death rates.^1,2^ We also focus on SUDs rather than substance use, as SUDs indicate the severity of substance use.

## Methods

### Population

The present study used deidentified data from the 2020 National Study on Drug Use and Health (NSDUH). Data were derived from N = 26,084 adult participants, representing 240 million adults weighted, and N = 5,723 adolescent participants, representing 25 million adolescents, ages 12-17. The NSDUH collects data annually on non-institutionalized U.S. citizens aged 12 and older and uses a stratified, multistage probability sample to obtain representative cohorts. Survey collection procedures involve all household screenings and individual in-person interviews. However, pandemic-related issues led to slight changes in the 2020 NSDUH data collection methodology. Data collection from January to March 2020 was as usual, but data collection paused, and new web-based data collection resumed in October-December to avoid in-person contact.^29^ Due to pandemic-related changes to the NSDUH, the study’s data source, a direct comparison of the year 2020 to previous years is not recommended; as such, we focus solely on the year 2020.^29^ Further information regarding the methodology of the NSDUH is available from SAMHSA.^30^

### Measures

#### Suicidality

Separate measures were administered to adults (aged 18+) and adolescents (aged 12-17). In adults, suicidal ideation, planning, and attempts were assessed using the following questions: “At any time in the past 12 months, that is from [DATEFILL] up to and including today, did you seriously think about trying to kill yourself?” “During the past 12 months, did you make any plans to kill yourself?” and “During the past 12 months, did you try to kill yourself?.” In adolescents, suicidal ideation, planning, and attempts were assessed using the following questions: “Did you think about killing yourself?,” “Did you make a plan to kill yourself?” and “Did you make a suicide attempt or try to kill yourself?” These were evaluated as binary variables in our analysis with no aggregation across items.

#### Substance Use Disorders (SUDs)

The NSDUH uses the Diagnostic Statistical Manual fifth edition (DSM-5)^31^ to identify SUDs. Individuals are categorized as having a SUD if they meet 2 out of 11 criteria that reflect cravings and other indicators of dependence on a substance and behaviors arising from substance misuse.^29,31^ In this study, participants categorized as having a SUD met the DSM-5 criteria for one or more substances.

#### Sociodemographic variables

In addition to the measures on suicidality, we evaluated key sociodemographic variables in our adult and adolescent samples and their correlation with the aforementioned suicidality measures. The variables include sex/gender (male, female), race/ethnicity (Non-Hispanic White, Black, Asian, Multiracial, Native American, Native Hawaiian, and Hispanic), age (delineated by adult age groups, 18-65+ and adolescent ages, 12-17), poverty level (living in poverty, income up to 2-times the federal poverty threshold, income more than 2 times the federal poverty threshold), educational attainment (less than high school diploma, high school diploma, some college or more), area of residence (large metro, small metro, nonmetro), criminal justice involvement (past 12-month probation or parole), and having one or more SUDs (yes, no).

### Data analysis

Separate logistic regressions were performed for adults and adolescents to assess the association of substance use disorders, as determined by DSM-5 criteria, and suicidal thoughts and behaviors, with separate models for the variables for ideation, planning, and attempts. The survey used separate question sets and variables in adults and adolescents to assess depressive symptoms, with the adult question set assessing past 12-month symptoms and the adolescent question set assessing the lifetime presence of symptoms. Multivariate logistic regression models assessed the association between suicidal thoughts and behaviors, substance use disorders, and gender in adolescent and adult cohorts. To address the possibility of multicollinearity, we examined the variation inflation factors (VIFs) for each variable in each of the 6 models. All VIFs were lower than 2, indicating that multicollinearity was not present.

## Results

### Sociodemographic characteristics of adult sample

Among the adult survey participants, 48% were male, and 52% were female. 63% identified as White, 12% identified as Black, 0.6% identified as Native American/Alaska Native, 0.3% identified as Native Hawaiian/Other Pacific Islander, 6% identified as Asian, and 16% identified as Hispanic (Table 1). Around 11% of adults in the sample had less than a high school diploma, 27% had at least a high school diploma, and 61% had some college-level education or a college degree. The age range and distribution of the sample included 13% 18-25 years of age, 16% 26-34 years of age, 25% 35-49, 24% 40-64, and 22% 65 years of age or older.

**Table 1.** Sample characteristics.

Sociodemographic variables	Adult sample Weighted N = 241 951 019 (Unweighted N = 26 084), %	Adolescent sample Weighted N = 24 982 702 (Unweighted N = 5723), %
*Sex*		
Females	125 216 988 (14 374), 52%	12 259 148 (2778), 49%
Males	116 743 030 (11 710), 48%	12 723 554 (2945), 51%
*Education*		
Less than high school	27 078 956 (2115), 11%	—
High school diploma	66 188 855 (5357), 27%	—
Some college	73 473 317 (8001), 30%	—
College	75 209 890 (10 611), 31%	—
*Age (Adults)*		
18-25 years old	31 795 375 (7522), 13%	—
26-34 years old	38 759 874 (5482), 16%	—
35-49 years old	59 400 993 (7167), 25%	—
50-64 years old	58 639 109 (3014), 24%	—
65 or older	53 355 669 (2899), 22%	—
*Age (Adolescents)*		
12 years old	—	3 740 718 (934), 15%
13 years old	—	4 713 234 (1068), 19%
14 years old	—	4 309 775 (1019), 17%
15 years old	—	4 200 917 (934), 17%
16 years old	—	4 175 533 (927), 17%
17 years old	—	3 842 525 (841), 15%
*Race*		
Non-Hispanic White	153 210 533 (17 552), 63%	12 820 007 (3218), 51%
Non-Hispanic Black	28 717 780 (2280), 12%	3 128 555 (588), 13%
Non-Hispanic Native American	1 359 393 (211), 0.6%	235 798 (67), 1%
Non-Hispanic Native Hawaiian/Other Pacific Islander	791 923 (106), 0.3%	30 723 (20), 0.1%
Non-Hispanic Asian	14 175 917 (1486), 6%	1 862 353 (311), 7%
Non-Hispanic Multiracial	4 324 024 (920), 2%	597 447 (351), 2%
Hispanic	39 371 449 (3529), 16%	6 307 819 (1168), 25%
*Poverty level*		
Living in poverty	35 036 686 (3683), 14%	4 677 512 (896), 19%
Income up to 2X Federal poverty threshold	43 478 700 (4391), 18%	4 682 192 (1056), 19%
Income more than 2X Federal poverty threshold	163 435 633 (18 010), 68%	15 622 998 (3771), 62%
*Area of residence*		
Large metro	131 551 941 (11 663), 54%	14 102 096 (2514), 56%
Small metro	73 872 258 (9791), 31%	7 600 660 (2103), 30%
Nonmetro	36 526 819 (4630), 15%	3 279 946 (1106), 13%
*Criminal justice involvement*		
Past year parole or probation	3 223 283 (354), 1%	—
None	239 112 325 (25 853), 99%	—
*Substance use disorder*		
Yes	35 336 896 (4632), 15%	1 438 607 (360), 6%
No	206 614 122 (21 452), 85%	23 544 095 (5363), 94%
*Suicidality in past 12 months*		
Suicidal ideation	11 602 049 (1791), 5%	3 369 571 (783), 13%
Planned suicide	3 015 886 (511), 1%	1 469 211 (363), 6%
Attempted suicide	1 110 518 (196), 0.5%	993 204 (223), 4%

Poverty level and area of residence among our sample varied. Around 14% of the adult sample lived in poverty; 18% had incomes up to twice the federal poverty level, while most participants (68%) had incomes more than twice the federal poverty level. Moreover, most participants lived in a large metropolitan area (54%), followed by small metro areas (31%), and a minority lived in nonmetro areas (15%). In addition, SUDs and criminal justice involvement were minimal in our sample. Around 15% of the adult participants had at least 1 SUD, and only 1% was on probation or parole in the past 12 months (1%). Regarding suicidality, 5% reported suicidal ideation, 1% reported making a suicide plan, and 0.5% endorsed a suicide attempt (Table 2). Therefore, in 2020, approximately 12 million U.S. adults (5%) seriously considered suicide, 3 million adults (1.2%) made a suicide plan, and 1 million adults (0.5%) attempted suicide.

**Table 2.** Multivariable logistic regressions assessing correlates of suicidality for adults and adolescents in the U.S. (2020).

Socio-demographic variables	Adults (18+ years)			Adolescents (12-17 years of age)		
	Suicidal ideation aOR (95% CI)	Suicide planning aOR (95% CI)	Attempted suicide aOR (95% CI)	Suicidal ideation aOR (95% CI)	Suicide planning aOR (95% CI)	Attempted suicide aOR (95% CI)
*Sex*						
Females vs males	**1.20 (1.00, 1.45)**	1.29 (0.94, 1.77)	**1.91 (1.14, 3.22)**	**3.40 (2.58, 4.47)**	**3.17 (2.20, 4.57)**	**4.85 (2.92, 8.04)**
*Race*						
Non-Hispanic Black vs. Non-Hispanic White	**0.63 (0.43, 0.92)**	0.65 (0.37, 1.15)	0.65 (0.30, 1.37)	0.88 (0.58, 1.34)	1.20 (0.65, 2.18)	0.88 (0.43, 1.78)
Non-Hispanic Native American vs. Non-Hispanic White	0.81 (0.30, 2.15)	0.80 (0.31, 2.07)	3.22 (0.73, 14.22)	**5.88 (1.05, 32.84)**	0.93 (0.22, 3.84)	0.68 (0.14, 3.36)
Non-Hispanic Native Hawaiian/Other Pacific Islander vs. Non-Hispanic White	0.58 (0.20, 1.65)	0.53 (0.08, 3.31)	1.05 (0.14, 8.10)	0.50 (0.054, 4.52)	1.78 (0.18, 17.31)	2.52 (0.25, 25.53)
Non-Hispanic Asian vs. Non-Hispanic White	**0.52 (0.34, 0.77)**	**0.18 (0.08, 0.41)**	**0.36 (0.14, 0.94)**	0.66 (0.38, 1.15)	1.01 (0.46, 2.24)	0.55 (0.15, 2.08)
Non-Hispanic Multiracial vs. Non-Hispanic White	1.28 (0.83, 1.98)	1.00 (0.49, 2.04)	1.22 (0.50, 3.00)	**2.15 (1.13, 4.07)**	1.36 (0.56, 3.28)	1.00 (0.37, 2.75)
Hispanic vs. Non-Hispanic White	**0.67 (0.51, 0.88)**	**0.55 (0.35, 0.86)**	0.67 (0.35, 1.28)	0.97 (0.66, 1.42)	0.94 (0.56, 1.60)	1.10 (0.63, 1.95)
*Age (adults)*						
26-34 years old vs. 18-25 years old	**0.63 (0.51, 0.77)**	**0.38 (0.27, 0.55)**	**0.34 (0.19, 0.61)**	—	—	—
35-49 years old vs. 18-25 years old	**0.43 (0.35, 0.54)**	**0.37 (0.24, 0.56)**	**0.22 (0.11, 0.43)**	—	—	—
50-64 years old vs. 18-25 years old	**0.33 (0.24, 0.46)**	**0.20 (0.12, 0.35)**	**0.14 (0.05, 0.37)**	—	—	—
65 or older vs. 18-25 years old	**0.19 (0.12, 0.30)**	**0.03 (0.01, 0.12)**	**0.01 (0.00, 0.06)**	—	—	—
*Age (adolescents)*						
13 years old vs. 12 years old	—	—	—	**1.98 (1.16, 3.37)**	0.94 (0.44, 2.04)	1.02 (0.40, 2.61)
14 years old vs. 12 years old	—	—	—	1.57 (0.92, 2.68)	1.39 (0.64, 3.02)	1.16 (0.44, 3.05)
15 years old vs. 12 years old	—	—	—	**2.73 (1.64, 4.53)**	**2.44 (1.19, 5.00)**	2.45 (0.98, 6.17)
16 years old vs. 12 years old	—	—	—	**2.84 (1.67, 4.81)**	**2.25 (1.10, 4.61)**	2.63 (0.99, 7.02)
17 Years old vs. 12 years old	—	—	—	**2.17 (1.24, 3.80)**	1.70 (0.82, 3.54)	1.30 (0.49, 3.43)
*Poverty level*						
Living in poverty vs. income more than 2X federal poverty threshold	1.25 (0.96, 1.63)	**1.62 (1.06, 2.45)**	**1.99 (1.06, 3.75)**	0.84 (0.58, 1.22)	**0.55 (0.32, 0.97)**	1.13 (0.62, 2.04)
Income up to 2X federal poverty threshold vs. income more than 2X federal poverty threshold	**1.30 (1.00, 1.68)**	1.23 (0.85, 1.79)	1.52 (0.84, 2.77)	0.99 (0.67, 1.44)	1.21 (0.73, 2.01)	**2.09 (1.20, 3.64)**
*Education*						
High school diploma vs. less than high school	1.13 (0.75, 1.70)	0.91 (0.51, 1.65)	1.09 (0.54, 2.21)	—	—	—
Some college vs. less than high school	1.43 (0.96, 2.15)	0.92 (0.52, 1.64)	0.73 (0.36, 1.45)	—	—	—
College vs. less than high school	1.02 (0.67, 1.56)	0.54 (0.28, 1.03)	0.42 (0.16, 1.11)	—	—	—
*Area of residence*						
Large metro vs. nonmetro	1.01 (0.78, 1.33)	1.21 (0.79, 1.85)	0.97 (0.53, 1.78)	1.23 (0.87, 1.75)	0.91 (0.58, 1.42)	0.99 (0.54, 1.79)
Small metro vs. nonmetro	1.11 (0.85, 1.45)	1.08 (0.72, 1.62)	0.81 (0.44, 1.49)	1.01 (0.69, 1.48)	0.74 (0.47, 1.19)	1.09 (0.59, 2.01)
*Criminal justice involvement*						
Past year parole or probation vs. none	1.12 (0.64, 1.95)	1.12 (0.48, 2.61)	2.38 (0.79, 7.18)	—	—	—
*Substance use disorder*						
Yes vs. no	**3.94 (3.19, 4.86)**	**3.09 (2.25, 4.25)**	**3.77 (2.29, 6.19)**	**3.69 (2.47, 5.51)**	**5.14 (3.25, 8.13)**	**5.27 (2.91, 9.53)**

Bold = significant at p-value < 0.05.

### Sociodemographic characteristics of the adolescent sample

Among the sample of adolescent survey participants, 51% were male, and 49% were female (Table 1). 51% identified as White, 13% identified as Black, 1% identified as Native American/Alaska Native, 0.1% identified as Native Hawaiian/Other Pacific Islander, 7% identified as Asian, 25% identified as Hispanic, and 2% identified with more than 1 race (Table 1). Among the adolescents, 15% were 12 years old, 19% were 13 years old, 17% were 14 years old, 17% were 15 years old, 17% were 16 years old, and 15% were 17 years old (Table 1).

Poverty level and area of residence among our adolescent sample were similar to our adult sample. Around 19% of the adolescent sample lived in poverty, 19% had family incomes up to twice the federal poverty level, while most adolescents (62%) had more than twice the federal poverty level. In addition, most adolescents lived in a large metropolitan area (56%), followed by small metro areas (30%), and a small percentage lived in nonmetro areas (13%).

SUDs were less prevalent in the adolescent sample than in the adult sample, yet suicidality was more prevalent in the adolescent than in the adult sample. Around 6% of all adolescent participants had a substance use disorder, and 13% reported suicidal ideation, 6% reported making a suicide plan, and 4% endorsed a suicide attempt (Table 2). This suggests that in 2020, around 3.3 million (13%) adolescents seriously considered suicide, 1.2 million (6%) adolescents made a suicide plan, and 1 million adolescents (4%) attempted suicide.

### Multivariable regression assessing correlates of suicidal ideation (seriously considering suicide)

**Figure 1.**
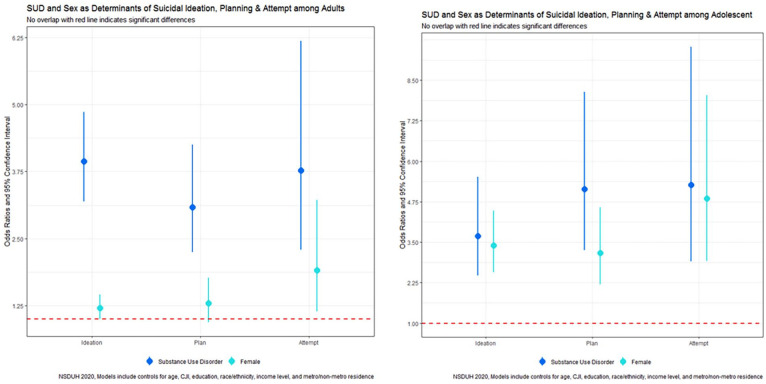
Suicidality by sex/gender among adults and adolescents.

**Figure 2.**
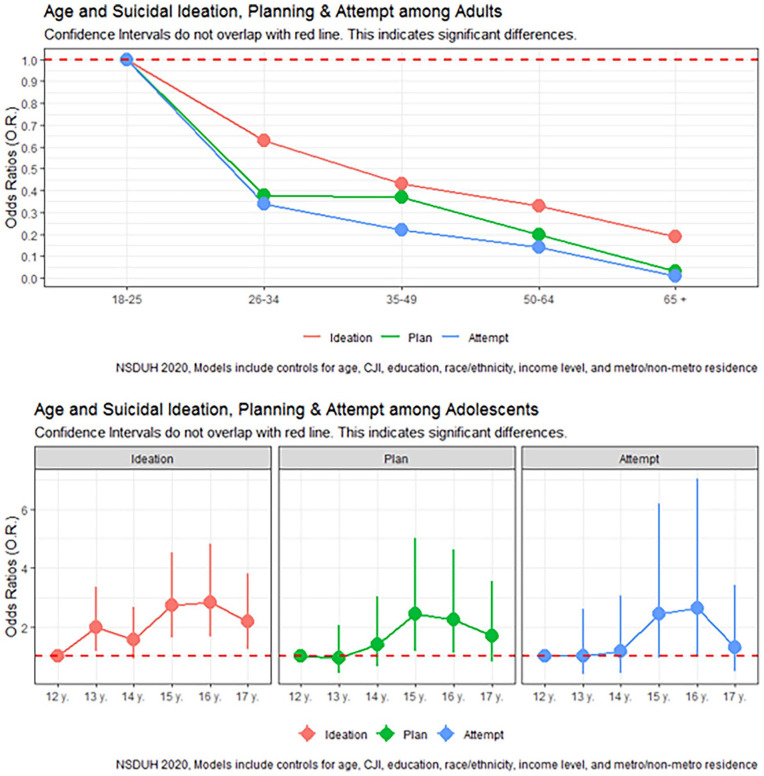
Suicidality by age among adults and adolescents.

In our multivariable logistic regression assessing suicidal ideation, several sociodemographic characteristics were significant correlates. Among U.S. adults, sex was significantly linked with suicidal ideation, with females having a 20% higher likelihood of suicidal ideation than men (aOR 1.20, 95% CI: 1.00, 1.45) (Figure 1, Table 2). Poverty level was also a significant correlate, with individuals with incomes up to twice the federal poverty level 30% more likely to have suicidal ideation in 2020 than those with incomes more than twice the federal poverty level (aOR 1.30, 95% CI: 1.00, 1.68). Interestingly, those living in poverty did not have a significantly increased likelihood of suicidal ideation than those with the highest incomes. SUDs were strongly correlated with suicidal ideation; those with at least 1 SUD were nearly 4 times more likely to seriously consider suicide (aOR = 3.94, 95% CI: 3.19, 4.86). Conversely, Non-White race/ethnicity and older age were correlated with decreased odds of suicidal ideation. Specifically, Non-Hispanic Black adults (aOR 0.63, 95% CI: 0.43, 0.92), Non-Hispanic Asians (aOR: 0.52, 95% CI: 0.34, 0.77), Hispanic adults (aOR: 0.67, 95% CI: 0.51, 0.88) were around 30%-50% less likely to report suicidal ideation than Non-Hispanic White individuals. Adults aged 26-34 (aOR 0.63, 95% CI: 0.51, 0.77), 35-49 (aOR 0.43, 95% CI: 0.35, 0.54), 50-64 (aOR 0.33, 95% CI: 0.24, 0.46), and 65 or older (aOR 0.19, 95% CI: 0.12, 0.30) were less likely to seriously consider suicide than those aged 18-25 years (Figure 2, Table 2).

Among U.S. adolescents, girls were over 3 times more likely to report suicidal ideation than boys (aOR = 3.40, 95% CI: 2.58, 4.47). Unlike adults, boys and girls from racially minoritized groups were likelier to report suicidal ideation than Non-Hispanic White individuals. Adolescents identifying as Native Americans (aOR = 5.88, 95% CI: 1.05, 32.84) and Non-Hispanic Multiracial (aOR = 2.15, 95% CI: 1.13, 4.07) were 2-6 times more likely to report suicidal ideation compared to Non-Hispanic White adolescents. Moreover, increasing adolescent age was typically linked with increased odds of suicidal ideation. Adolescents aged 13 (aOR = 1.98, 95% CI: 1.16, 3.37), 15 (aOR = 2.73, 95% CI: 1.64, 4.53), 16 (aOR = 2.84, 95% CI: 1.67, 4.81), and 17 (aOR = 2.17, 95% CI: 1.24, 3.80) were more likely to report suicidal ideation than those 12 years of age. No significant differences in suicidal ideation between 12 and 14 years were observed. In addition, adolescents with at least 1 SUD were over 3.5 times more likely to report suicidal ideation than those without SUDs (aOR = 3.69, 95% CI: 2.47, 5.51).

### Multivariable regression assessing correlates of planning a suicide attempt

In our multivariable logistic regression assessing planning a suicide attempt, poverty level, and SUDs were linked with increased odds. Individuals living in poverty were 60% more likely to plan suicide in 2020 than those with incomes more than twice the federal poverty level (aOR 1.62, 95% CI: 1.06, 2.45). Similarly, those with SUDs were 3 times more likely to plan a suicide attempt suicide (aOR = 3.09, 95% CI: 2.25, 4.25) than those without SUDs. Non-White race/ethnicity and older age were significantly correlated with decreased odds of planning a suicide attempt. Specifically, Non-Hispanic Asians (aOR: 0.18, 95% CI: 0.08, 0.41), Hispanic adults (aOR: 0.55, 95% CI: 0.35, 0.86) were around 80%-50% less likely to report planning a suicide attempt than Non-Hispanic White individuals. Adults aged 26-34 (aOR 0.38, 95% CI: 0.27, 0.55), 35-49 (aOR 0.37, 95% CI: 0.24, 0.56), 50-64 (aOR 0.20, 95% CI: 0.12, 0.35), and 65 or older (aOR 0.03, 95% CI: 0.01, 0.12) were less likely to seriously consider suicide than those aged 18-25 years.

Among U.S. adolescents, females were over 3 times more likely to report planning a suicide than boys (aOR = 3.17, 95% CI: 2.20, 4.57). Moreover, older adolescents were more likely to report planning a suicide attempt. Adolescents aged 15 (aOR = 2.44, 95% CI: 1.19, 5.00) and 16 (aOR = 2.25, 95% CI: 1.10, 4.61) were likelier to report planning a suicide attempt than those 12 years of age. Adolescents with SUDs were over 5 times more likely to report planning a suicide than individuals without SUDs (aOR = 5.14 95% CI: 3.25, 8.13), while adolescents living in poverty were less likely to plan a suicide (aOR: 0.55, 95% CI: 0.32, 0.97). No significant differences in planning a suicide between those aged 13, 17, and 14 years compared to those aged 12 years of age nor adolescents from racially/ethnically minoritized groups compared to Non-Hispanic White adolescents were observed.

### Multivariable regression assessing correlates of attempted suicide

Our multivariable logistic regression assessing correlates of suicide attempts, that females were nearly twice as likely to attempt suicide than men (aOR 1.91, 95% CI: 1.14, 3.22). Similar to suicide planning, poverty level, and SUDs increased the odds of attempting suicide. Individuals living in poverty were twice as likely to plan a suicide attempt in 2020 than those with incomes more than twice the federal poverty level (aOR 1.99, 95% CI: 1.06, 3.75). Similarly, those with SUDs were nearly 4 times more likely to plan a suicide attempt suicide (aOR = 3.77, 95% CI: 2.29, 6.19) than those without SUDs. Non-Hispanic Asian race and older age were significantly correlated with decreased odds of planning a suicide attempt. Specifically, Non-Hispanic Asians (aOR: 0.36, 95% CI: 0.14, 0.94) were around 60% less likely to report planning a suicide attempt than Non-Hispanic White individuals. Adults aged 26-34 (aOR 0.34, 95% CI: 0.19, 0.61), 35-49 (aOR 0.22, 95% CI: 0.11, 0.43), 50-64 (aOR 0.14, 95% CI: 0.05, 0.37), and 65 or older (aOR 0.01, 95% CI: 0.00, 0.06) were less likely to report attempting suicide than those aged 18-25 years.

Among U.S. adolescents, girls were nearly 5 times more likely to report attempting suicide than boys (aOR = 4.85, 95% CI: 2.92, 8.04). Adolescents with SUDs were also over 5 times more likely to report attempting suicide than individuals without SUDs (aOR = 5.27 95% CI: 2.91, 9.53), and adolescents with household incomes up to twice that of the federal poverty level were more likely to attempt suicide (aOR: 2.09, 95% CI: 1.20, 3.64). No significant differences in suicide attempts by age, race/ethnicity, and area of residence were observed.

## Discussion

Suicides are a leading cause of death among those who have SUDs, particularly due to the disinhibition involved with intoxication.^18^ As such, we used large, nationally representative data to examine the relationship between SUDs and suicidal thoughts and behaviors in adolescents and adults during 2020, a year where substance use and global uncertainty increased.^2^ Our results illustrated that adults with SUDs had a 3- to 4-fold greater risk of suicidal thoughts and behaviors than those without SUDs. Similarly, adolescents with SUDs had a 4- to 5-fold greater risk of suicidal behaviors than adolescents without SUDs. Current research from Canada found that adults who use drugs were nearly twice as likely to have suicidal ideation during the pandemic than those who did not report drug use.^32^ In addition, research from international contexts corroborates our finding that adolescents who use drugs were significantly more likely to report suicidal ideation and self-harm than adolescents abstaining from drug use. Although, the magnitude was much less than this study’s findings.^33^ The results from this current study and that of the international settings suggest a need for comprehensive interventions focusing on comorbid SUDs and mental health issues. Particularly, integrated cognitive behavioral therapy that addresses both substance use and suicidality has been shown to reduce symptoms and should be provided to patients with these co-occurring conditions.^34,35^ Research on integrated care for dual diagnoses suggests this model increases treatment adherence and reduces logistical and financial barriers compared to treating separate conditions.^34,35^

We also found that suicidality was stronger for females than males and particularly salient among adolescent females, and adolescents, in general, experience higher rates of suicidal ideation than adults. These findings confirm previous research examining gender and age differences in suicide.^24,36^ Yet, the research by Ivey-Stephenson et al^24^ found higher rates of suicidality among their sample of adolescents (19% suicidal ideation, 16% planning a suicide attempt, and 9% with previous suicide attempts) in 2019 than found in this current study. However, this current study used the NSDUH survey while Ivey-Stephenson et al^24^ used the Youth Risk Behavior Surveillance Survey (YRBS). In addition, our sample of adolescents was younger than the study mentioned above (ages 12-17 vs ages 14-18), and it is expected that older adolescents would have a higher likelihood of suicidality. For Ivey-Stephenson et al^24^, significant increases in suicide attempts were only seen for high school students in 12th grade, those aged 17 and 18.

Moreover, we found that racially/ethnically minoritized individuals among U.S. adults reported less suicidality than Non-Hispanic White adults during the pandemic. Contrastingly, other research has found that among U.S. adults, racially/ethnically minoritized individuals in the United States were more likely to experience suicidal ideation.^2,37^ These discrepancies may be attributed to the differences in sampling techniques between the studies. Non-Hispanic Multiracial and Native American adolescents were significantly more likely to have suicidal ideation, though no significant racial/ethnic differences in suicide planning and attempts were evident. In addition, racial/ethnic differences have been noted in increasing suicidality for White and Black boys.^24^ These results have implications for developing and implementing harm reduction interventions, targeting those at the highest risk of negative outcomes, such as adolescent females and racially minoritized groups. As previously discussed, most forms of psychopathology increase the risk of suicidal ideation and attempts^19,21^; however, since these risks are greater in those with comorbid SUDs and psychopathology, targeted interventions and screening should be considered based on the various factors that can affect risk, including age and gender. Additionally, given the relationship that has been noted between suicidal ideation and state lockdown policies,^10^ consideration should be given to evaluating and mitigating such risks if these lockdown measures are reimplemented.

Many of the factors that are associated with the development of psychopathology are likely involved in the relationship between the enactment of lockdown policies and an exacerbation of mental health symptoms, specifically loneliness, social isolation, reduced social support, and limited access to mental health services in the community and school settings.^10,38,39^ Many youths receive mental health services in schools,^40^ as such, school closures would reduce access to school-based mental health services,^41^ increasing barriers to care and potentially delaying early intervention, diagnosis, and treatment. Multiple analyses have found increases in mental health symptoms among youth due to school closures and the shift to remote schooling,^42,43^ with the risk amplified further among vulnerable and marginalized populations. Primary preventive interventions should, therefore focus on reducing established risk factors such as social isolation and loneliness and, given the magnitude of the effect among adolescents, reducing the duration of school closures or increasing access to school-based mental health services during closures much as feasible. Further, there are some school-based interventions^44,45^ that have shown potential benefits in reducing suicidal thoughts and behaviors; efforts should be made to adapt existing evidence-based preventive and screening strategies to modified schooling environments, such as virtual or hybrid models.

### Limitations and strengths

Our study should be viewed in the context of its limitations. We are careful not to have any causal language or imply that the COVID-19 pandemic influenced significant associations, as this cannot be conclusively said. The NSDUH data collection methods were tailored to the changing pandemic landscape in the year 2020; as such, comparisons between the year 2020 and preceding years may not be appropriate, as any changes may reflect methodological differences between years rather than concrete changes. In addition, the NSDUH survey used the DSM-5 instead of the DSM-IV criteria to define SUDs; this new methodology reflects a higher sensitivity for SUDs than the previous methodology.^46^ The NSDUH relies on participant self-report data, introducing the possibility of recall and social desirability bias. Moreover, we could not directly examine the presence of serious mental health in our study as it was too collinear with suicidality and produced unstable estimates. One meta-analysis examining case-control psychological autopsy studies found that the risk for suicide is 9 times and 7 times higher in those with major depression and substance use disorders, respectively.^10^

Furthermore, because the survey is administered only to civilian and non-institutionalized populations, it excludes those institutionalized in prison or hospital settings and active-duty military members. Lastly, the NSDUH survey is for individuals 12 years and older; children younger than 12 are not included in the survey and thus cannot be included in our analyses. As such, these results may not be generalizable to those specific populations. This study benefits from the use of a large, nationally representative dataset.

## Conclusion

Individuals with substance use disorders were significantly more likely to consider, plan, and attempt suicide than those without SUDs in 2020. This relationship was even more pronounced among adolescents than adults and more for women than men. Overall, our findings support screening for suicidality among patients who use drugs and integrated interventions to address both conditions. In developing primary prevention strategies and harm reduction programs, consideration should be given to differential effects by age, sex/gender, and other social factors on the risk of negative psychosocial outcomes. Evidence-based harm reduction strategies should be implemented proactively to reduce the future morbidity associated with pandemic-related effects on SUDs, suicidal thoughts and behaviors, and psychopathology more broadly.
